# Sex steroid hormone levels associated with dopamine D_2/3_ receptor availability in people who smoke cigarettes

**DOI:** 10.3389/fnbeh.2023.1192740

**Published:** 2023-06-09

**Authors:** Yasmin Zakiniaeiz, Ralitza Gueorguieva, MacKenzie R. Peltier, Terril L. Verplaetse, Walter Roberts, Sherry A. McKee, Kelly P. Cosgrove

**Affiliations:** ^1^Department of Psychiatry, School of Medicine, Yale University, New Haven, CT, United States; ^2^Department of Biostatistics, School of Public Health, Yale University, New Haven, CT, United States; ^3^Psychology Service, Veterans Affairs Connecticut Healthcare System, West Haven, CT, United States; ^4^Yale Positron Emission Tomography (PET) Center, School of Medicine, Yale University, New Haven, CT, United States; ^5^Department of Radiology and Biomedical Imaging, School of Medicine, Yale University, New Haven, CT, United States

**Keywords:** sex steroid hormones, dopamine, tobacco smoking, sex, positron emission tomography (PET)

## Abstract

**Introduction:**

Sex differences exist in tobacco smoking. Women have greater difficulty quitting smoking than men. Tobacco smoking is driven by the reinforcing effects of nicotine, the primary addictive component in cigarettes. Nicotine binds to nicotinic acetylcholine receptors, facilitating dopamine release in striatal and cortical brain regions. Dysregulated dopamine D_2/3_ receptor signaling in the dorsolateral prefrontal cortex (dlPFC) is associated with cognitive deficits such as impairments in attention, learning, and inhibitory control that impede quit attempts. Sex steroid hormones, such as estradiol and progesterone, influence drug-taking behaviors, through dopaminergic actions, suggesting that their influence may explain sex differences in tobacco smoking. The goal of this study was to relate dlPFC dopamine metrics to sex steroid hormone levels in people who smoke and healthy controls.

**Methods:**

Twenty-four (12 women) people who smoke cigarettes and 25 sex- and age-matched controls participated in two same-day [^11^C]FLB457 positron emission tomography scans, one before and one after amphetamine administration. D_2_R availability (*BP*_ND_) at baseline and after amphetamine administration was calculated. On the same day, plasma samples were collected for the analysis of sex steroid hormone levels: estradiol, progesterone, and free testosterone.

**Results:**

Women who smoke had trending lower levels of estradiol than their sex-matched counterparts. Men who smoke had higher levels of estradiol and trending higher levels of free testosterone than their sex-matched counterparts. Among women only, lower estradiol levels were significantly associated with lower pre-amphetamine dlPFC *BP*_ND_.

**Discussion/conclusion:**

This study demonstrated that lower estradiol levels are associated with lower dlPFC D_2_R availability in women which may underlie difficulty resisting smoking.

## Introduction

Sex differences exist in tobacco smoking. Women become addicted more quickly after exposure (Thorner et al., 2007), experience greater health consequences (US Department of Health Human Services, 2014), are less responsive to the first line of treatment (nicotine replacement therapies) (Fattore et al., 2008), and are more likely to relapse after abstinence (Fattore et al., 2008). Tobacco smoking is driven in part by the reinforcing effects of nicotine, the primary addictive component in cigarettes. In the brain, nicotine binds to and activates nicotinic acetylcholine receptors, facilitating dopamine release in striatal and cortical regions (Benowitz, 2010; Cosgrove et al., 2015) *via* the mesolimbic and mesocortical dopamine pathways, respectively. The mesolimbic (“reward”) dopamine pathway drives the reinforcing effects of tobacco smoking, while the mesocortical (“goal-directed”) dopamine pathway—including the dlPFC—is critical for inhibitory control (Wu et al., 2013). Dysregulated dopamine D_2/3_ receptor (D_2_R) signaling in dlPFC is associated with cognitive deficits, such as impairments in attention, learning, working memory, and inhibitory control (Arnsten et al., 2015), that are known to impede quit attempts. Literature has shown that sex steroid hormones, such as estradiol and progesterone, influence drug-taking (including nicotine) behaviors, through dopaminergic actions. This suggests that sex steroid hormone influences reward and cognition may partially explain sex differences in tobacco smoking and the greater vulnerability in women. We have previously shown that women (vs. men) who smoke have greater dlPFC dopamine deficits. However, the relationship between these dopamine deficits and sex steroid hormone levels has not yet been examined.

Cigarette smoking has been associated with adverse reproductive outcomes including infertility, subfecundity, early menopause, and menstrual disorders (Department of Health Human Services, 2001). Cigarette smoke contains known toxicants to the reproductive system (Mattison and Thorgeirsson, 1978) and promotes estrogen deficiency or hypoestrogenism (Baron et al., 1990). Among women, heavy smoking is associated with shorter and more variable menstrual cycle lengths with the shorter menstrual cycle lengths occurring primarily in the follicular phase (Windham et al., 1999). While the biological mechanisms of these findings are not known, the literature suggests that the effects of smoking on hormone secretion and metabolism are mainly mediated by the pharmacological action of nicotine and its metabolite, cotinine (Marom-Haham and Shulman, 2016). Furthermore, a growing body of data, including epidemiological data, suggests that these effects may be non-linear [reviewed in Marom-Haham and Shulman (2016)]. More studies are needed to fully understand the complex pathobiology of cigarette smoking and its hormonal impact.

A preclinical study has shown that sex steroid hormones are involved in the acquisition of, motivation for, and reinstatement of drug taking. Female rats acquire drug self-administration in an operant conditioning chamber more rapidly than male rats (Lynch, 2006), which has been shown to be primarily due to circulating ovarian hormones in females; estradiol exposure enhances the rate of acquisition of drug taking (Lynch et al., 2001; Jackson et al., 2006; Hu and Becker, 2008). Female rats exhibit a higher breaking point to obtain drugs on a progressive ratio schedule than males, suggesting a greater drug motivation (Roberts et al., 1989; Cummings et al., 2011). This effect is enhanced by estradiol treatment in ovariectomized rats (Becker and Hu, 2008). With respect to reinstatement or relapse-like behavior, estradiol enhances while progesterone attenuates reinstatement in females (Fuchs et al., 2005; Feltenstein and See, 2007), through modulation of dopamine (Castner et al., 1993; Cummings et al., 2014). Because progesterone may attenuate some of the subjective effects of drugs of abuse and thereby decrease drug intake (Evans and Foltin, 2006; Jackson et al., 2006), sex steroid hormones may help direct treatments for addiction (Lynch et al., 2002).

The emerging body of literature on the interactions of dopamine and sex steroid hormones in addiction to nicotine has predominantly focused on ovarian hormones. For example, in castrated rats, estrogen differentially modulated nicotine-induced dopamine release in the striatum, enhancing dopamine release in female rats and reducing dopamine release in male rats (Dluzen and Anderson, 1997). In humans, cyclical changes in ovarian hormones over the course of the menstrual cycle alter sensitivity to the reinforcing effects of nicotine through interactions with the dopamine system (Carpenter et al., 2006). Taken together, this suggests that nicotine may interact with sex steroid hormones within the context of the dopamine system to impact the vulnerability of women to cigarette smoking. However, this topic is critically understudied, and this has not been directly tested.

Our group previously showed that dlPFC D_2_R availability was significantly lower in people who smoke than healthy controls and that drug-induced dlPFC dopamine release (using amphetamine, a robust dopamine probe) is blunted in women who smoke compared to men who smoke and healthy control women (Zakiniaeiz et al., 2019). We also showed that lower dlPFC D_2_R availability was associated with poorer cognitive function (Zakiniaeiz et al., 2022). However, we did not examine whether dlPFC D_2_R availability before and after amphetamine administration was related to sex steroid hormones. The goal of this study was to relate dopamine metrics to sex steroid hormone levels in people who do and do not smoke. Based on dopamine-related findings from our group and prior literature, we hypothesized that in women, lower dlPFC D_2_R availability before and after amphetamine administration would be related to lower estradiol and higher progesterone levels. Based on limited research, as an exploratory secondary analysis, we also hypothesized that lower dlPFC D_2_R availability before and after amphetamine administration would be related to lower testosterone levels.

## Materials and methods

### Subjects

Twenty-four (12 women) people who smoke and 25 age- and sex-matched healthy controls (12 women) participated in two same-day [^11^C]FLB457 PET scans, one scan before (“baseline”) and the second scan 3 h after amphetamine administration (0.4–0.5 mg/kg, PO), at peak amphetamine levels. Peak amphetamine levels relate to peak extracellular dopamine (Narendran et al., 2014) and provide maximum sensitivity for us to detect differences in *BP*_ND_ between pre- and post-amphetamine conditions. The [^11^C]FLB457 PET (Zakiniaeiz et al., 2019) and cognitive task (Zakiniaeiz et al., 2022) data from these subjects have been previously published. Written informed consent for all study procedures, approved by the Yale Human Investigation Committee and the Yale-New Haven Hospital Radiation Safety Committee, was obtained from all subjects prior to participation. The study adhered to the Protection of Human Subjects of Research and Ethical Principles and Guidelines.

Subject screening procedures included a physical exam, electrocardiogram, blood tests, and urine toxicology. Subjects had no history of significant major medical disorders and did not meet the DSM-IV criteria for current or past psychiatric or substance use disorder diagnosis (except nicotine dependence for people who smoke). People who smoke were required to have been smoking cigarettes daily for at least 1 year. On intake day, tobacco smoking status was confirmed by spirometry to measure carbon monoxide (CO) levels >11 parts per million (ppm) and by urine samples to measure cotinine—the primary metabolite of nicotine levels >150 ng/ml (NicAlert cotinine test strips; Nymox Pharmaceutical). On scan day, overnight abstinence was required and confirmed by CO levels < 10 ppm or ≤ 50% of their intake level. All women subjects were required to have a negative pregnancy test on intake day and on PET scan day prior to radiotracer administration.

### Sex steroid hormone analysis

On PET scan day, plasma samples were collected in all subjects prior to the first scan for analysis of sex steroid hormone levels: estradiol, progesterone, and free testosterone. All plasma collection took place around 9 a.m., to control for fluctuations in hormone levels. Menstrual cycle phase was not controlled on scan day. Plasma samples were available for 44 of the 49 subjects; five subjects (three women who smoke and two men who smoke) did not provide blood samples due to difficulty in obtaining blood or PET scan timing logistics. Plasma samples were stored in −80°C freezers and analyzed by the Yale Center for Clinical Investigations using the FDA-registered Alpco Serum ELISA kits for direct quantitative determination by enzyme immunoassay. Valid test ranges and sensitivity respectively were as follows: progesterone (0.3–60 ng/ml; 0.1 ng/ml), estradiol (20–3,200 pg/ml; 10 pg/ml), and free testosterone (0.1–60 pg/ml; 0.1 ng/ml). Sex steroid hormone levels were included in the analysis if they were within three standard deviations from the subgroup mean. Following this standard outlier detection method, only one data point was excluded—a healthy control woman with a free testosterone level of 15 ng/ml. Because estradiol and progesterone data were skewed, the data were log-transformed to better approximate normality.

### Imaging data acquisition, processing, and analysis

PET data acquisition and analysis were previously described (Zakiniaeiz et al., 2019). In brief, the high-affinity D_2/3_ radioligand [^11^C]FLB457 was injected intravenously as a bolus over 1 min by a computer-controlled pump. Emission data were collected for 90 min using an ECAT EXACT HR+ (Siemens/CTI, Knoxville, TN, USA), following a 6-min transmission scan for attenuation correction. A structural T1 magnetic resonance imaging (MRI; Trio, Siemens Medical Systems, Erlangen, Germany) was acquired for anatomical localization of the *a priori* dlPFC region-of-interest (ROI). The dlPFC was our *a priori* ROI because of its primary role in the mesocortical pathway and its impairment in cognitive disorders in animal and human studies (Arnsten et al., 2015), including studies from our group (Zakiniaeiz et al., 2019, 2022).

Sinograms were reconstructed with filter-back projection using a SUN workstation, with all corrections into a sequence of 27 frames. Motion correction was performed on dynamic image data by registering each frame to a summed early frame using a six-parameter mutual information algorithm (Viola and Wells Iii, 1997) (FMRIB's Linear Image Registration Tool version 3.2). PET summed images were smoothed at a 3 × 3 × 3 voxel FWHM Gaussian filter. Image dimensions and voxel size were 128 × 128 × 63 and 2.06 × 2.06 × 2.43 mm^3^, respectively. The final reconstructed image resolution was ~6 mm FWHM.

Each MR image was normalized to Montreal Neurological Institute (MNI) space using an affine linear plus non-linear registration (Bioimage Suite 2.5), to extract the ROIs (Zakiniaeiz et al., 2019). The dlPFC was defined by combining the frontal superior, frontal mid, and frontal inferior triangularis corresponding to Brodmann's areas of 9 and 46 (Zakiniaeiz et al., 2019). PET data were fitted with the simplified reference tissue model (SRTM) using the cerebellum as a reference region and IDL software to estimate *BP*_ND_ (the ratio at equilibrium of specifically bound radioligand to that of the concentration of nondisplaceable radioligand in tissue) for pre- and post-amphetamine scans, as previously validated and described (Zakiniaeiz et al., 2019). *BP*_ND_ is an index of D_2_R availability that is proportional to the number of available binding sites.

### Statistics

Sex steroid hormone levels were compared between women who smoke and their sex-matched counterparts and men who smoke and their sex-matched counterparts using independent samples *t*-tests.

Linear mixed models were used to assess the effect of sex steroid hormone levels on pre- and post-amphetamine *BP*_ND_ by smoking status, using identical models to our prior report (Zakiniaeiz et al., 2019). The within-subjects factor was the effect of amphetamine (pre or post), and the between-subjects factor was smoking status (person who smokes or healthy control). We included estradiol as a continuous predictor in the model. Identical linear mixed models were performed with progesterone and free testosterone as the continuous predictors. We decided *a priori* to create sex-specific models to explore the hypothesized associations within each sex group, and we did not correct for multiple comparisons in our models. Our continuous predictor variables (estradiol, progesterone, and free testosterone) were mean-centered to aid the interpretation of coefficients. All two-, three-, and four-way interactions were included. Non-significant main effects and interactions were removed from the models in a stepwise fashion so that at each step, the models were hierarchically well formulated. An α-level of 0.05 was used for all main effect and interaction tests. All statistics were conducted using SPSS, version 28. Supplementary analyses examined the ratios of estradiol and progesterone as continuous variables.

## Results

### Subject characteristics

Groups were well matched on demographics, such as age and sex, as previously reported (Zakiniaeiz et al., 2019, 2022). On average ± SEM, women who smoke were 32 ± 3.5 years old, and healthy control women were 31 ± 3.0 years old (*p* = 0.89). Three out of 9 women who smoke and 6 out of 11 healthy control women reported taking hormonal contraceptives (*p* = 0.34). One woman who smoked (age 50 years old) and one healthy control woman (age 51 years old) reported being postmenopausal (*p* = 0.81). Including age, hormonal contraceptives, and menopausal status in the statistical models did not affect the results. Women and men who smoked were well matched in smoking characteristics; women smoked 12.8 ± 1.5 cigarettes per day for 13.5 ± 2.0 years and men smoked 13.6 ± 1.5 cigarettes per day for 16.1 ± 1.9 years (*p* = 0.70 and *p* = 0.36, respectively).

### Sex steroid hormones

**Figure 1:**
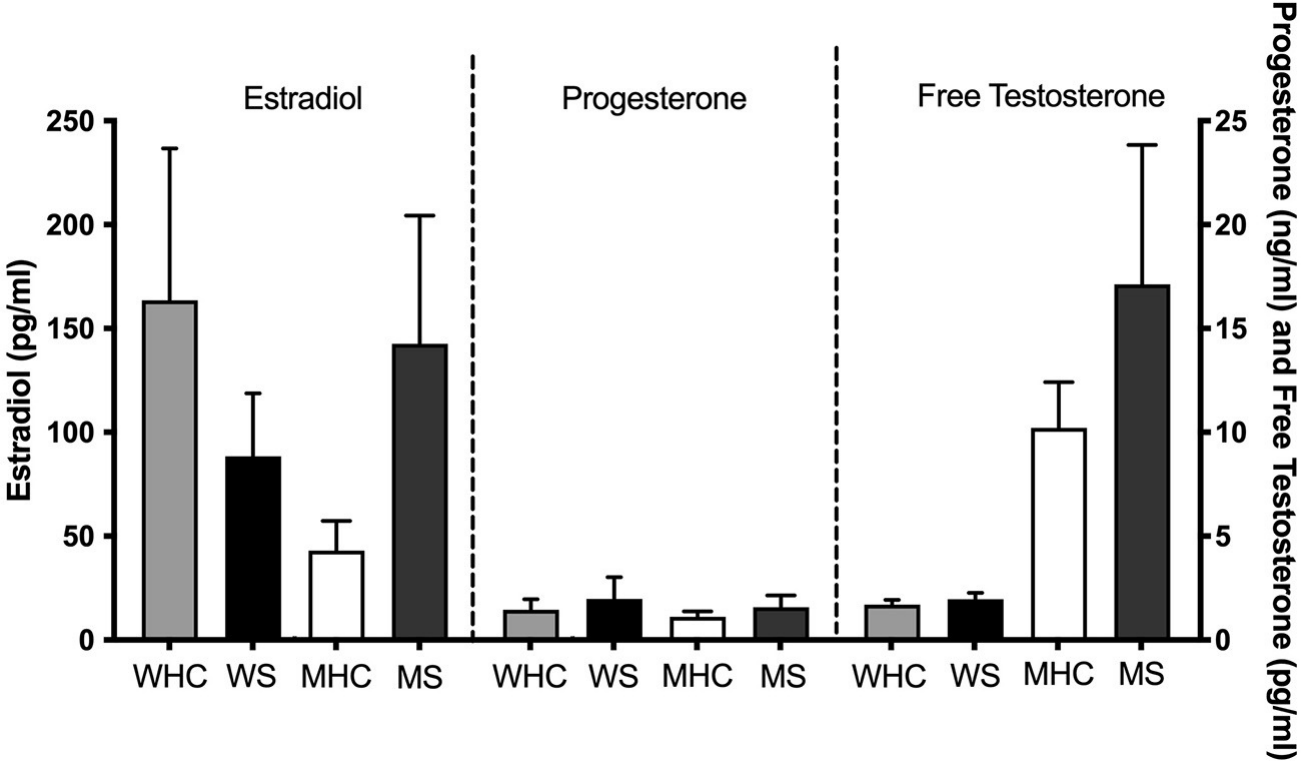
Mean sex steroid hormone levels for four subgroups. Estradiol, progesterone, and free testosterone levels shown for healthy control women (WHC; *n* = 12 for estradiol and progesterone; *n* = 11 for testosterone), women who smoke (WS; *n* = 9), healthy control men (MHC; *n* = 13), and men who smoke (MS; *n* = 10). WS have trending lower levels of estradiol (*p* = 0.09) than their sex-matched counterparts. Men who smoke have significantly higher levels of estradiol (*p* = 0.02) and trending higher levels of free testosterone (*p* = 0.08) than their sex-matched counterparts. SEM is shown.

Mean sex steroid hormone levels for the four subgroups are shown in Figure 1. Ranges are shown in Supplementary Table S1. Independent samples *t*-tests revealed that women who smoke have trending lower levels of estradiol (*t* (30) = 1.38, *p* = 0.09) than sex-matched counterparts. Men who smoke have significantly higher levels of estradiol (*t* (21) = 2.28, *p* = 0.02) and trending higher levels of free testosterone (*t* (23) = 1.43, *p* = 0.08) than sex-matched counterparts.

### Dopamine and sex steroid hormones

**Figure 2:**
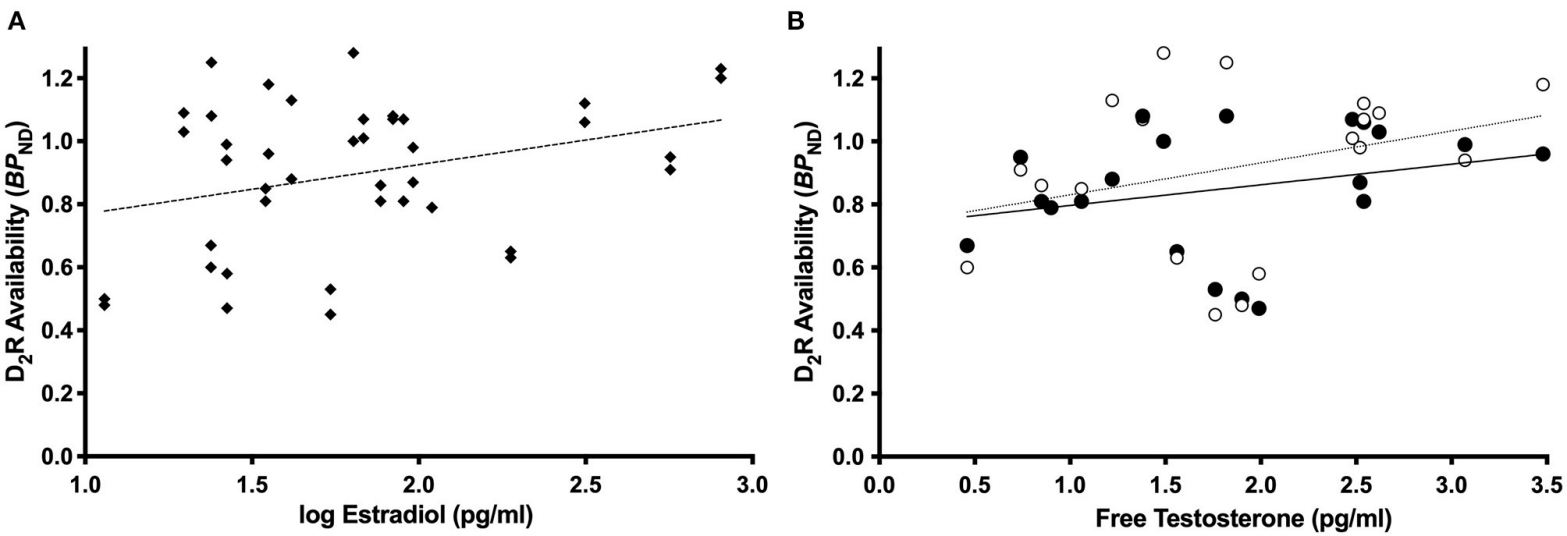
Dopamine receptor availability and sex steroid hormone relationships. Among women only (*n* = 12 WHC; 9 WS), estradiol was related to dlPFC *BP*_ND_, *p* = 0.02 **(A)**. Among women only (*n* = 11 WHC; 9 WS), free testosterone was related to the effect of amphetamine on *BP*_ND_, *p* = 0.08 **(B)**, such that there is a trending stronger relationship pre-amphetamine compared to post-amphetamine. Open circles and dotted line = pre-amphetamine; closed circles and solid line = post-amphetamine.

Consistent with our previous report in the full sample (Zakiniaeiz et al., 2019), all linear mixed effects models revealed a significant effect of amphetamine and interaction of amphetamine by smoking status on *BP*_ND_, *p* < 0.05. For women only, the linear mixed effects model with estradiol revealed a significant main effect of estradiol on *BP*_ND_, *F* (1.18) = 6.17, *p* = 0.02 (Figure 2A). Lower estradiol was associated with lower *BP*_ND_ (slope = 0.22; standard error = 0.09). For women only, the linear mixed effects model with progesterone did not reveal a significant main effect of progesterone on *BP*_ND_, *F* (1.18) = 1.54, *p* = 0.23, inconsistent with our hypothesis. For women only, the linear mixed effects model with free testosterone revealed a trending interaction of amphetamine by free testosterone on *BP*_ND_, *F* (1.17) = 3.47, *p* = 0.08 (Figure 2B). There is a positive relationship between free testosterone and *BP*_ND_. There is a trending stronger relationship pre-amphetamine (slope = 0.12; standard error = 0.70), compared to post-amphetamine (slope = 0.06; standard error = 0.56), but none of the slopes are significantly different from zero. Linear mixed models in men only did not reveal any statistically significant effects. Supplementary linear mixed models examining the ratios of estradiol and progesterone did not reveal any statistically significant effects.

## Discussion

There were three main findings from this study: (1) women and men who smoke have different levels of sex steroid hormones compared to sex-matched counterparts, (2) for women only, lower levels of estradiol were significantly associated with lower dlPFC D_2_R availability, and (3) for women only, trending lower levels of free testosterone were associated with lower dlPFC D_2_R availability pre-amphetamine relative to post-amphetamine. In men, sex steroid levels were not significantly associated with dopamine metrics. A previously published study from our group showed that people who smoke tobacco had significantly lower dlPFC D_2_R availability compared to healthy controls (Zakiniaeiz et al., 2019) and that lower dlPFC D_2_R availability was associated with poorer cognitive function (Zakiniaeiz et al., 2022). This study extends these findings by relating dlPFC D_2_R availability to sex steroid hormone levels in people who smoke tobacco, providing a better understanding of the influence of sex steroid hormones on the dopamine system (Zakiniaeiz and Cosgrove, 2020).

Lower estrogen levels have previously been observed in women who smoke (Westhoff et al., 1996), consistent with the trend observed in our study. Our findings are also consistent with previous human research reporting that men who smoke have higher concentrations of testosterone than their healthy control counterparts (English et al., 2001; Allen et al., 2002; Muller et al., 2003; Shiels et al., 2009; Liu et al., 2021). One study found that men who formerly smoked do not show this effect, suggesting that the association between testosterone and smoking is reversible (Shiels et al., 2009). Some studies have shown a dose-response relationship between the number of cigarettes smoked and testosterone levels (Tamimi et al., 2001; Allen et al., 2002) while others do not (Handa et al., 1997; Svartberg and Jorde, 2007). In contrast to our study, one study showed that male mice exposed to cigarette smoke have lower levels of testosterone relative to sex-matched controls (Yardimci et al., 1997). The relationship and mechanism between cigarette smoking and testosterone levels remains unclear. One possible mechanism is that cigarette smoke and nicotine may act as aromatase inhibitors, thus reducing the conversion of testosterone to estradiol. However, inconsistent with this theory, we also observed higher estradiol levels in men who smoke. Similar findings were previously reported (Svartberg and Jorde, 2007; Shiels et al., 2009), while other studies found no relationship between testosterone levels and smoking (Handa et al., 1997; English et al., 2001; Muller et al., 2003). Furthermore, our results show that women who smoke have lower levels of estradiol than their sex-matched counterparts while men who smoke have higher levels of estradiol than their sex-matched counterparts. This sexual dimorphism may be confounded by menstrual cycle phase which was not controlled in our study. Our findings and the current literature suggest that cigarette smoking alters sex steroid hormone levels, but more studies are needed to uncover the biological mechanism.

Among women, lower levels of estradiol were associated with lower dlPFC D_2_R availability, consistent with our hypothesis. In line with this finding, a preclinical study showed that there is a sexually dimorphic effect of estradiol on the D_2_R, where estradiol rapidly downregulates D_2_ binding in females but not in males (Bazzett and Becker, 1994). Prior literature predominantly focused on the influence of ovarian hormone fluctuations on dopamine and the mesolimbic and nigrostriatal, but not on the mesocortical dopamine pathway. The relationship between menstrual phase-related fluctuations in estrogen and/or progesterone on dopamine has produced mixed results (Wong et al., 1988; Nordström et al., 1998; Munro et al., 2006). The current study serves as a snapshot of the relationship between dopamine receptor levels and sex steroid hormone levels rather than ovarian hormone fluctuations, in the mesocortical dopamine pathway. More studies are needed to determine whether there is a direct relationship between tobacco smoking, estradiol levels, and dopamine receptor levels, how this affects dopamine receptor functioning, and whether it is reversible with smoking cessation.

Among women, we also observed a trend such that lower levels of free testosterone were associated with lower dlPFC D_2_R availability pre-amphetamine compared to post-amphetamine, consistent with our hypothesis. Literature on the relationship between dopamine and testosterone is limited as most observations have been reported in ovarian hormones. One study examining the effects of sex steroid neonatal exposure to sex steroid hormones showed that a single injection of testosterone in 1-day-old female rat pups showed an intermediate non-significant effect on morphine-induced dopamine release, whereas estradiol significantly enhanced dopamine release (Bonansco et al., 2018). Another study using the Study of Women Across the Nation (SWAN) dataset found that among perimenopausal women who engage in heavy alcohol use, those with higher levels of testosterone were less likely to reduce their drinking (Peltier et al., 2020). In our study, associations between sex steroid hormone levels and dopamine receptor levels were only observed in women suggesting that hormones may differently modulate dopamine in women vs. men. More studies are needed to untangle the complicated relationship between D_2/3_Rs and sex steroid hormones, especially testosterone, which is critically understudied in women.

The strengths of this study include the relatively large PET dataset and plasma measures of sex steroids. This study also has some limitations that can be addressed in future studies. First, while the sample size allowed for systematic examinations between subgroups, missing plasma data in people who smoke reduced the sample size, which may have impacted our ability to detect the effect of smoking status on the relationship between dopamine and sex steroids. Second, data included in these analyses included variables (e.g., sex steroid hormone levels and dopamine levels) that were evaluated at only one-time point. Furthermore, because menstrual cycle phase was not controlled, we observed variability in sex steroid hormone levels which made it difficult to detect subgroup differences in sex steroid levels. However, literature has shown that the examination of sex steroid hormone levels vs. menstrual cycle phase more precisely quantifies the impact of dynamic changes in hormone levels throughout the cycle (Weinberger et al., 2015). This is a critical future direction (Allen et al., 2008; Mazure et al., 2010). Future studies should also focus on understanding the underlying biological mechanisms by which smoking may alter sex steroids and sex steroids may alter dopamine. Taken together, our findings suggest that cigarette smoking alters sex steroid levels and in women, sex steroids influence dopamine receptor levels which may impede abstinence from cigarette smoking.

## Data availability statement

The raw data supporting the conclusions of this article will be made available by the authors, without undue reservation.

## Ethics statement

The studies involving human participants were reviewed and approved by the Yale Human Investigation Committee and the Yale-New Haven Hospital Radiation Safety Committee. The patients/participants provided their written informed consent to participate in this study.

## Author contributions

YZ: formal analysis, data curation, project administration, methodology, and writing—original draft. RG: formal analysis, methodology, and writing—reviewing and editing. MRP, TLV, and WR: methodology and writing—reviewing and editing. SAM: supervision, methodology, and writing—reviewing and editing. KPC: supervision, conceptualization, methodology, funding acquisition, and writing—reviewing and editing. All authors contributed to the article and approved the submitted version.
