# Supplementary material for: Sex steroid hormone levels associated with dopamine D2/3 receptor availability in people who smoke cigarettes
Source: Front Behav Neurosci. 2023 Jun 9;17:1192740. doi: 10.3389/fnbeh.2023.1192740 (PMC10288103; doi:10.3389/fnbeh.2023.1192740)
Supplement: Supplementary file 1 [file Table_1.DOCX]

Supplemental Material

Zakiniaeiz et al., Sex steroid hormone levels associated with dopamine D_2/3_ receptor availability in people who smoke cigarettes.

**Table S1: Hormone Levels for all 4 subgroups.** Estradiol, Progesterone, and Free Testosterone Levels shown for women healthy controls (WHC), women who smoke (WS), men healthy controls (MHC), and men who smoke (MS).

|  | **WHC** (N=12 or 11) | | | **WS** (N=9) | | | **MHC** (N=13) | | | **MS** (N=10) | | |
| --- | --- | --- | --- | --- | --- | --- | --- | --- | --- | --- | --- | --- |
|  | Mean | Min | Max | Mean | Min | Max | Mean | Min | Max | Mean | Min | Max |
| Estradiol (pg/ml) | 163.6 | 11.4 | 803.8 | 88.5 | 19.8 | 314.4 | 43.0 | 3.0 | 179.8 | 142.6 | 29.4 | 671.3 |
| Progesterone (ng/ml) | 1.5 | 0.2 | 6.5 | 2.0 | 0.2 | 10.2 | 1.1 | 0.1 | 3.7 | 1.6 | 0.2 | 5.4 |
| Free Testosterone (pg/ml) | 2.8 | 0.7 | 14.8 | 2.0 | 0.5 | 3.1 | 10.2 | 4.7 | 34.6 | 17.1 | 4.1 | 75.3 |
